# Preferences for private health insurance in China: A discrete choice experiment

**DOI:** 10.3389/fpubh.2022.985582

**Published:** 2022-09-06

**Authors:** Nuo Chen, Jing Bai, Stephen Nicholas, Elizabeth Maitland, Jialong Tan, Jian Wang

**Affiliations:** ^1^Dong Fureng Institute of Economic and Social Development, Wuhan University, Beijing, China; ^2^Australian National Institute of Management and Commerce, Sydney, NSW, Australia; ^3^Newcastle Business School, University of Newcastle, Newcastle, NSW, Australia; ^4^School of Management, University of Liverpool, Liverpool, United Kingdom; ^5^School of Public Health, Wuhan University, Wuhan, China; ^6^Center for Health Economics and Management at the School of Economics and Management, Wuhan University, Wuhan, China

**Keywords:** private health insurance, Huimin Insurance, discrete choice experiment, health insurance preferences, China

## Abstract

**Introduction:**

There is limited evidence on the sustainability and optimal design of China's private health insurance market, especially from the demand-side. With the increasing medical cost burden on both patients and the social security system, policy makers need data on potential clients' demand for private health insurance.

**Methods:**

A discrete choice experiment was conducted to explore potential clients' preferences for a type of government-involved private supplementary health insurance, Huimin Insurance, in China. A mixed logit model was used to evaluated participants' preferences for six attributes. Willingness to pay, subgroup analysis and interaction effects were estimated based on the initial model.

**Results:**

Among the 947 participants, 883 (93.2%) were aged 18 to 59 years and 578 (61.0%) were female. Participants had a strong preference for government involvement, extensive benefit packages, high reimbursement ratio and compensation for pre-existing conditions. With respect to the attribute of deductible, participants were indifferent between the level of CNY15,000 and CNY18,000 but had strong and significant preference for the level of CNY15,000 than CNY20,000. The premium was significantly correlated with a decline in the utility of PHI.

**Conclusions:**

All attributes had a significant impact on participants' preference for Huimin Insurance. Providing a reference point for the development of private health insurance in China, our results inform the optimal design of PHI, especially Huimin Insurance's products.

## Introduction

Private health insurance (PHI) refers to health insurance schemes provided by commercial insurance companies or not-for-profit agencies (1). In developed countries, PHI offers comprehensive insurance coverage, including countries with universal health coverage where PHI complements and supplements public health insurance schemes. Complementary PHI provides coverage for services not covered by public schemes, while supplementary PHI provides extra coverage for services that are only partly covered by public schemes.

Since the early 2010s, China has achieved universal health coverage through the provision of social health insurance (SHI), with over 95% of the population covered by basic health insurance. China's SHI consists of two basic schemes, the Urban Employee Basic Medical Insurance (UEBMI) (2) and Urban-Rural Residents Basic Medical Insurance (URRBMI) (3). UEBMI was launched in 1998 and financed by employer and employee contributions. URRBMI merged the Urban Residents Basic Medical Insurance (covering the urban unemployed, children and students) and the Newly Cooperative Medical Scheme (covering rural residents), which is financed by government subsidies and individual premiums. SHI in China plays a vital role in providing basic health insurance, particularly for the vulnerable populations, such as the poor and those living in rural areas. But SHI's overall level of protection remains low. According to the 2020 China National Health Accounts Report, CNY4.1 trillion medical costs were incurred, but only 50% of medical expenditures were reimbursed by SHI. An estimated 44% of China's CNY4.1 trillion medical costs were out-of-pocket (OOP) expenses paid by patients themselves. In contrast, PHI plays a negligible role in the financing of health expenditures in China, with just 5% (CNY 0.2 trillion) of the total medical expenditures covered by PHI.

Given the financial medical cost burden on patients (4, 5) and on the social security system (6, 7), China encouraged the development of PHI (8, 9) in recent years. In February 2020, the State Council of China proposed to build a multi-layered medical security system with basic medical insurance as the mainstay and medical aid as the base, private health insurance, charitable donations, and mutual medical assistance as supplements by 2030. However, PHI has long been criticized for its low payout rates, estimated to be only 36.5% of medical expenses in 2020, and playing an insignificant role in the financing of medical treatment. PHI is provided mainly by for-profit commercial insurance companies and purchased primarily by higher-income groups. Price discrimination, short duration, strict reimbursement conditions and failure to provide ongoing coverage explain the relatively low enrollment rates, barely reaching 4% of the population (10, 11).

To deal with dysfunctional PHI and to lower the financial burden on those with high medical costs, a type of government-involved, private supplementary insurance was introduced by Huimin Insurance in 2015 and expanded in 2020. As a model for PHI, Huimin Insurance features low insurance premiums, high level of services coverage and no threshold for participation. Typically, a Huimin Insurance scheme covers co-payments and deductibles not reimbursed by SHI as well as services not covered by SHI. The main objective of the insurance is to reduce the financial burden on enrollees due to treatment of severe and chronic diseases, such as cancer. Since 2020, 177 Huimin Insurance products in cooperation with local government have been launched in 244 cities and 28 provinces, with 140 million participants, collecting about CNY14 billion in premiums.

As a model of public-private partnership, Huimin is often operated at city level, since most SHI schemes are managed at city level in China, where local governments participate in Huimin Insurance and other private schemes to varying degrees. Most local governments supervise the operation of PHI and many cooperate with private insurers by sharing local SHI reimbursement data. Local governments sometimes endorse particular PHI products, help design the benefit package and provide a one-stop settlement of payments, where SHI reimbursements and PHI reimbursements are processed through one application. In return, private insurers promise increased payout rates and limit the maximum profit margin from the plan (usually between 5 and 10%), above which any profit should be reimbursed to participants. This local government cooperation is typical for Huimin Insurance products.

A typical challenge most PHI face is the operational sustainability. A survey of Huimin Insurance schemes in the marketplace indicate that the average enrollment rate is merely above 5%, which significantly affects the insurers' ability to manage the risk and cover all operating costs. In addition, the design of uniform premiums and low threshold for participation for all residents have the risk of adverse selections (12, 13) from the elderly and those who are ill. Those low-risk individuals may be phasing out of insurance because they do not receive a commensurate return. Moreover, the generous benefit packages such as expensive cancer drugs and high ceilings of imbursements in most schemes put the insurers at risk of moral hazard (14, 15) from both participants and providers which could ultimately drive up the costs to the schemes. To avoid potential excessive losses, most Huimin insurance schemes have adopted the strategy of reimbursing only OOP expenses for hospitalization services within SHI catalog and a small portion of specialty drugs costs outside SHI catalog in their benefit package design, setting deductible at a high level and excluding or lowering the compensating for major pre-existing conditions. As a result, such design of the insurance schemes narrows the scope of coverage and reduces the pay-out rate, limiting their attractiveness and enrollment rates. How to balance the relationship between premiums, benefit package and enrollment is crucial for the long-term sustainability of Huimin Insurance. Preference exploration for insurance attributes is crucial to the design of suitable PHI products, which makes discrete choice experiments (DCE) a powerful analytical tool to address insurance design.

Surprisingly, research on PHI in China is sparse in this respect. Existing studies mostly focused on PHI's current level of development (16, 17), its impact on equity in healthcare (9), ex-ante and ex-post moral hazard from consumers (18) and issues relating to the regulation of its operations (18). However, there has been no quantitative study of the optimal design of PHI from the consumer perspective. Addressing this lacuna, our study is the first discrete choice experiment to evaluate prospective clients' preferences, along with willingness to pay (WTP) and population heterogeneity, for Huimin Insurance as an example of PHI. Our DCE results inform the optimal design of PHI, especially Huimin Insurance, providing a reference point for the PHI market in China.

## Methods

Discrete choice experiments (19–21) are an effective and reliable method used in the health field to examine stated preferences over hypothetical alternative scenarios (22–30). Each scenario comprises various attributes (government involvement, premium, benefit package, deductible, reimbursement ratio and compensation for pre-existing conditions), and each attribute has different levels. Willingness to pay and the probability analysis were based on the discrete choice model. Four main steps comprised our DCE: identifying relevant attributes and associated levels; designing experiments and developing questionnaires; carrying out the experiments and collecting data, and analyzing the data. Data were collected through an offline survey in October 2021 in a sample of residents in Shiyan, Hubei province. Data analysis was performed from October 2021 to July 2022. We used DCE to assess how the product features influence people's preferences for Huimin Insurance, and then explore which sociodemographic characteristics affect participants' preferences.

### Study setting

Shiyan is an industrial and mountainous city in the Hubei province of China. The city has a population of 3.21 million, with more than a third of its population living under the national poverty alleviation standard in 2014. Like many other cities in China, the SHI in Shiyan mainly consists of UEBMI and URRBMI, where the local ceilings for UEBMI reimbursements are CNY120 thousand and CNY100 thousand for URRBMI reimbursements. Despite the local government gradually adjusting its SHI policy to raise the ceilings and increase the reimbursement rates, SHI patients face high OOP medical expenses, particularly those who suffer from serious illness, such as cancer. According to the local administrative data, the ratio of OOP expenses to total medical costs for an inpatient service was 24% for the UEBMI and 32% for the URRBMI in 2020. Given these large OOP medical expenditures, there is a huge demand for alternative financing mechanisms, especially PHI.

### Attribute and level identification

To determine the DCE attributes and levels, a two-stage approach was adopted, involving a document review and in-depth interviews with health and insurance experts (31–33). First, we performed a document review of Huimin Insurance products released before May 1 2021. From the document analysis, we found homogeneity in current products, mostly focusing on eight attributes: government involvement, premium, benefit package, deductible, reimbursement ratio, ceiling, compensation for pre-existing conditions and value-added services. Second, we conducted a quadrilateral in-depth interview with two insurance executives, two policy makers and three academic experts. Through extensive discussions with local social health insurance officials and the analysis of the local SHI claims data, we have gathered information on disease burden, economic burden of diseases and OOP payments for treatment on local residents. Based on this, we adjusted the corresponding attributes and levels to better reflect the local situations. To make the DCE manageable for participants, we controlled the number of attributes (34), setting the ceiling at a fixed level of CNY1 million and excluding the value-added services attribute. Experts pointed out that value-added services varied significantly between products and was not a key element determining people's preferences for PHI in China. The final six attributes, their definition and their levels are presented in Table 1.

**Table 1:** Characteristics of attributes and attribute levels.

**Attributes**	**Definition**	**Levels**
Government involvement^a^	Whether the government back Huimin Insurance	No Yes
Premium	Individual premium actually paid	39 CNY/year
		79 CNY/year
		119 CNY/year
		159 CNY/year
		199 CNY/year
Benefit package	Reimbursement coverage for claims	Basic: Out-of-pocket expenses for hospitalization services within social health insurance catalog
		Expanded 1: Out-of-pocket expenses for hospitalization services within social health insurance catalog + specialty drugs costs outside social health insurance catalog^b^
		Expanded 2: Out-of-pocket expenses for hospitalization services within social health insurance catalog + specialty drugs costs outside social health insurance catalog + out-of-pocket expenses for hospitalization services not covered by social health insurance
Deductible	Reimbursement thresholds for claims	15,000 CNY
		18,000 CNY
		20,000 CNY
Reimbursement ratio	Reimbursement proportion for claims	60%
		80%
		100%
Compensation for pre-existing conditions^c^	Whether the expenses for pre-existing conditions can be compensated	No Yes

a Government involvement meant promotion in official channels or premiums could be offset using funds from social health insurance personal accounts.

b Zero deductible for specialty drug costs.

c “Pre-existing conditions” meant any sickness, disease, injury, physical, mental or medical condition or physiological degradation, including congenital condition, that existed prior to the policy issuance date or the policy effective date, whichever was the earlier.

### Experimental design and questionnaire development

Given the attributes and their levels in Table 1, a full factorial design produced 540 (= 2^2^ × 3^3^ × 5^1^) hypothetical scenarios and 145,530 [(540×539)/2] pairwise choice tasks. A D-efficient design (35) was used to generate 13 manageable choice sets using Stata16.0. As shown by the example in Table 2, we adopted an unlabelled design and each choice set composed two options. An opt-out option was not incorporated into the DCE. The primary concern is that participants may choose the opt-out option because the choice helps to avoid making a difficult decision between alternative options, not because it can generate the highest utility (36). In addition, as our prime focus is the participants' relative preferences for different attributes, it is likely to be a hindrance for us to include an opt-out option as participants' choice of an opt-out option provide limited information (37). To check for internal consistency, a dominant choice set was placed in half of the questionnaire, with the alternative clearly better than the other attributes (38, 39). Each participant needed to make 14 DCE choices (the choice sets can be found in Supplementary material 1). Participants who failed the consistency test were excluded from the analyses.

**Table 2:** Example of choice set.

**Attributes**	**A**	**B**
Government involvement	No	Yes
Premium (CNY per year)	159	119
Benefit package	Out-of-pocket expenses for hospitalization services within social health insurance catalog	Out-of-pocket expenses for hospitalization services within social health insurance catalog + specialty drugs costs outside social health insurance catalog
Deductible (CNY)	18,000	20,000
Reimbursement ratio	60%	100%
Compensation for pre-existing conditions	No	Yes
**Which one do you prefer?**		

Before the final data collection, we conducted a pre-test on 20 Shiyan residents. Minor revisions were made to ensure the questionnaire was easy to understand. Based on the residents' feedback, we also improved the verbal interpretation of the attributes to ensure that participants had no cognitive difficulties in comprehension. The questionnaire was composed of two sections: personal background information (comprising age, sex, residence type, marital status, education level, chronic conditions, yearly income, SHI and other PHI) and DCE scenarios.

### Sampling and data collection

We collected data through face-to-face interviews during October 2021. A multi-stage stratified random sampling method was used to determine the sample. We first randomly selected one district in urban and rural areas in Shiyan and then randomly selected one or two streets in each district. Next, we randomly selected one or two communities in each street. Finally, we identified four urban sample sites and one rural sample site, with each containing approximately 200 households. The participants were restricted to those who had a local household registration or SHI and were aged 18 to 75.

Proposed by Orme and Johnson (40, 41), the formula for determining the minimum sample size for discrete choice experiments:


$$\begin{array}{r} N\geq \frac{500c}{t\times a} \end{array}$$

where c is the maximum number of levels of the included attributes, t is the number of choice sets, and a is the number of options in each choice set (excluding exit options) (42). Based on the above formula, the sample size should be no <500×5/(2×13) ≈96. We aimed to collect a minimum sample of 500 participants. To increase the response rate and ensure data quality, community public officials played an important role, by assisting the investigation group to identify sampled households, ensuring only one member of a sampled household was interviewed and providing interview space at the local community center. The community public officials also assisted the investigators to communicate with those participants who had a strong local accent during the DCE interviews. Overall, a total of 973 questionnaires were distributed and 947 valid questionnaires were completed, with an effective response rate of 97.3%.

### Statistical analysis

Based on the random utility theory (43), we utilized a mixed logit model to analyze the data, which allowed for potential preference heterogeneity (44). WTP expresses the relative monetary value that participants place on the private insurance attributes (45, 46). To calculate WTP, the yearly premium was treated as a continuous variable and the remaining attributes were coded as dummy variables. Subgroup analyses (47) and interaction effects (39) were also performed to explore the sociodemographic sources of preference heterogeneity. We carried out several simulations based on the sociodemographic characteristics of participants to explore the preference changes among different subpopulation. All analyses were conducted using Stata 16.0.

## Results

### Descriptive statistics

Table 3 summarizes the participants' characteristics. Among 947 participants, 883 (93.2%) were aged 18 to 59 years, 578 (61.0%) were female, and 784 (82.8%) were from urban areas. Most were educated [826 (87.2%)], married [785 (82.9%)] and without chronic diseases [717 (75.7%)]. Shiyan's per capita disposable yearly income for urban residents was CNY32,771 and CNY11,731 for rural residents, with 556 participants (58.7%) possessing annual income higher than the average level. Almost all the participants were covered by social health insurance [937 (98.9%)], with only 10 interviewees (1.1%) self-reporting as not having SHI, and 293 participants (30.9%) had previously purchased extra PHI.

**Table 3:** Participants' characteristics.

**Characteristic**	**No. (%) (*n* = 947)**
Sex	
Male	369 (39.0)
Female	578 (61.0)
Age, y	
18–59	883 (93.2)
60–75	64 (6.8)
Residence	
Rural	163 (17.2)
Urban	784 (82.8)
Marital status	
Widowed, unmarried, or divorced	162 (17.1)
Married	785 (82.9)
Education level	
Uneducated (<6 y)	121 (12.8)
Educated (≥6 y)	826 (87.2)
Chronic conditions	
Unknown	34 (3.6)
None	717 (75.7)
One of more	196 (20.7)
Yearly income	
Lower than Shiyan average	391 (41.3)
Higher/equal Shiyan average	556 (58.7)
Social health insurance	
None	10 (1.1)
URRBMI	366 (38.6)
UEBMI	571 (60.3)
Other private health insurance	
No	654 (69.1)
Yes	293 (30.9)

URRBMI, Urban-Rural Residents Basic Medical Insurance; UEBMI, Urban Employee Basic Medical Insurance.

### Preferences for PHI

Table 4 shows participants' preferences for PHI, where all attributes had a significant impact with expected signs. Four estimated standard deviations were significant in Table 4—having government involvement, expanded benefit package 2 and 100% reimbursement ratio and compensating for pre-existing conditions, indicating the existence of preference heterogeneity.

**Table 4:** Participants' preferences for private health insurance.

**Attributes**	**Mean (SE)**	**SD (SE)**	**Relative effect-change in loglikelihood**	**Order of importance**
Government involvement			27.26%	2
No	Reference	-		
Yes	0.707^***^ (0.048)	0.999^***^ (0.045)		
Premium, CNY/year	−0.004^***^ (0.003)	-	3.79%	5
Benefit package			19.23%	3
Basic	Reference	-		
Expanded 1	0.663^***^ (0.037)	−0.022 (0.107)		
Expanded 2	1.023^***^ (0.048)	0.555^***^ (0.053)		
Deductible, CNY			0.16%	6
15,000	Reference	-		
18,000	−0.003 (0.034)	−0.003 (0.083)		
20,000	−0.095^*^ (0.037)	−0.170 (0.128)		
Reimbursement ratio, %			39.53%	1
60	Reference	-		
80	0.667^***^ (0.050)	−0.008 (0.070)		
100	1.250^***^ (0.036)	0.904^***^ (0.050)		
Compensation for pre-existing conditions			10.04%	4
No	Reference	-		
Yes	0.446^***^ (0.037)	0.626^***^ (0.040)		

SE, standard error; SD, standard deviation; CNY, Chinese Yuan.

*** p < 0.001,

* p < 0.05.

From Table 4, our participants had a strong preference for PHI with government involvement (vs. No: β = 0.71, *p* < 0.001), extensive benefit packages (vs. basic) (expanded 1: β = 0.66, *p* < 0.001; expanded 2: β = 1.02, *p* < 0.001), high reimbursement ratio (vs. 60%) (80%: β = 0.67, *p* < 0.001; 100%: β = 1.25, *p* < 0.001) and compensation for pre-existing conditions (vs. No: β = 0.45, *p* < 0.001). With respect to the attribute of deductible, participants were indifferent between CNY15,000 and CNY18,000 (β = −0.003, *p* = 0.94) but had strong and significant preference for CNY15,000 compared to CNY20,000 (β = −0.10, *p* = 0.011). The premium was significantly correlated with a decline in the utility of PHI reflected in the negative coefficient (β = −0.004, *p* < 0.001).

Based on the relative effect-change in the log likelihood of the various PHI attributes from the basic model, we sorted the relative importance of PHI attributes in Table 4. The reimbursement ratio (39.5%) was found to have the greatest impact on participants' PHI choices, followed by government involvement (27.3%), benefit package (19.2%) and compensation for pre-existing conditions (10.0%). These four attributes cumulatively contributed 96.1% to the overall explanatory power of participants' choice. Premiums (3.8%) and deductible (0.2%) contributed the least to overall preferences for PHI.

### Willingness to pay

Table 5 shows WTP estimation for PHI. We found a clear willingness among participants regarding the reimbursement ratio. Participants were willing to pay CNY351.1 (95% CI, CNY286.0-CNY416.2) when the reimbursement ratio increased from 60% to 100%, and participants were willing to commit an extra CNY287.3 (95% CI, CNY229.9-CNY344.7) to top-up the benefit package from the basic to the comprehensive. When PHI received government involvement, participants were willing to pay an additional CNY198.6 (95% CI, CNY155.5-CNY241.6).

**Table 5:** Willingness to pay for private health insurance.

**Attributes**	**WTP (95% CI), CNY**
Government involvement	
No	Reference
Yes	198.6 (155.5, 241.6)
Benefit package	
Basic	Reference
Expanded 1	186.1 (150.4, 221.8)
Expanded 2	287.3 (229.9, 344.7)
Deductible, CNY	
15,000	Reference
18,000	−0.8 (−19.7, 18.1)
20,000	−26.7 (−48.0, −5.3)
Reimbursement ratio, %	
60	Reference
80	187.3 (152.0, 222.7)
100	351.1 (286.0, 416.2)
Compensation for pre-existing conditions	
No	Reference
Yes	125.3 (96.6, 154.1)

WTP, willingness to pay; CNY, Chinese Yuan.

### Subgroup analysis and interaction effects

Subgroup analysis for participants' sociodemographic characteristics revealed that there were some slight variations in preferences by subgroups, but overall preferences were similar in Supplementary material 2. Compared to the overall result, there existed differences only for the deductible. The preferences remained the same in sex and SHI subgroups. For the subgroup of age, the younger age group (age 18–59) preferred CNY15,000 deductible over CNY20,000 deductible while for the older group (age 60–75) the deductible was not sensitive. For those living in urban areas or married or educated or having chronic diseases or yearly income lower than Shiyan average or without extra PHI had the same preferences as the younger group.

Interaction terms were introduced to further test sources of heterogeneity. The results presented in Supplementary material 2 show that urban/having other PHI participants had higher preferences for compensating for pre-existing conditions than rural/without extra PHI participants. UEBMI participants placed more emphasis on a Huimin plan with government involvement and a comprehensive benefit package and educated participants also highly valued government involvement. Compared with those with lower Shiyan average incomes, participants with higher incomes attached more importance to the expanded benefit package 2. No subgroup showed a clear preference for 100% reimbursement ratio.

## Discussion

Using preferences for Huimin Insurance as a typical private insurance scheme, this is the first study exploring preferences for PHI in China from the demand-side. All attributes in the model had a significant impact on the participants' choices and were consistent with the principle of utility-maximization (48), with the reimbursement ratio, government involvement and benefit package as the top three attributes driving participants' preferences. The reimbursement level was the most decisive attribute for participants, indicating participants' strong desire to reduce the coinsurance rate.

Government-promoted insurance had a high participation rate because participants were more likely to accept PHI products out of trust in the government. The government inadvertently acts as a guarantor, which enhances the equity in access to health care (49). One further advantage of government support is that residents can have their premiums deducted from their individual health insurance accounts rather than from their personal bank account (50).

Third, the benefit package with comprehensive services was a major attribute. Preferences increased with the addition of specialty drugs and extra hospitalization expenses, revealing participants demand for coverage of medical procedures that could result in high costs. Similar to the prior DCE research on health insurance (51), our benefit package reflects participants' demand for health services outside those provided by compulsory state social health insurance and the diverse health status of residents caused by factors such as regional and wealth disparities (52).

One key finding was that the deductible as an attribute had the least weight. This was related to the product positioning of PHI as supplementary to China's SHI to prevent catastrophic health expenditure. In our study, PHI products had high deductibles ranging from CNY15,000 to CNY20,000, which means very few of participants would be eligible to make claims, unless they suffer from a serious disease. Considering the actuarial solvency of insurers (53) and existing levels of deductibles in Huimin insurance products across the country, this study set the attribute of deductible with three levels ranging from CNY15- 20,000, which provided limited fluctuations for the insured with huge medical costs.

It is worth mentioning that some WTP estimates have exceeded the upper limit of insurance premium levels. Many would argue that the actual participation rate would be high when the real product contains the attributes with high WTP, which is contradictory to what we have observed in the real world. The main reason for the deviation is that in addition to WTP, the actual participation of an insurance plan also depends on one's ability to pay (ATP) (54). ATP is often highly correlated with individual or household financial status (55). When a participant is from the low income group, even when they indicate a WTP for the product, they would most likely not participate in the insurance plan due to low ATP. Excluding opt-out in DCE may result in an overestimation of WTP (39). By focusing on measuring the relative preference over different attributes of PHI, the constraints did not affect our main conclusion (36).

The subsample analysis revealed that there was no significant difference on the preference over all attributes except for the deductible between different subgroups of the population. The preference for deductible can be explained by the adverse selection of those with higher health risks (56). A basic prediction of the insurance theory model is that insurance markets will be prone to adverse selection when consumers have private information about their exposure to loss (57, 58). Nyman (59) suggested that health insurance contracts are compensatory transactions, where consumers agree to forego the consumption of other goods and services to pay a premium when they are healthy. When consumers are ill, they receive an income transfer from those who are healthy when they access expensive medical services, which explains the incentive to purchase health insurance. Thus, vulnerable groups are often willing to pay more to get better insurance coverage with lower deductibles (18).

The interaction effects showed that the sociodemographic sources of preference heterogeneity. Residence types, income level, SHI schemes types and whether own other PHI are the factors of heterogeneity. Moreover, the higher quality groups (living in urban areas, higher income, UEBMI, having extra PHI) tend to long for better coverage of Huimin Insurance. The income effect raises the demand for health insurance (60) and raises the value of increased insurance benefits, which is consistent with the argument that risk aversion increases with the returns from universal health insurance (61). Knowing preference heterogeneity for Huimin Insurance help policymakers understand individual demand, which will facilitate coverage rate.

Our study provides several policy implications. The DCE results indicate that both local government and commercial insurance companies' efforts are indispensable for an attractive and sustainable healthcare product. The strong preference for the attribute of government participation generally reflects the public's lack of trust toward PHI in China. As the central government increasingly recognizes the importance of PHI in the financing of health expenditures in China, local governments should play an enabling and facilitator role in the development of PHI market, such as using tax incentives. For commercial insurance companies, a feasible product design is the key and should take account of consumers' risk appetite and local disease burdens. For example, specialty drug benefits of the insurance schemes can include drugs that are used to treat conditions with relatively high disease burden in the local area. Meanwhile, China has reformed its drug management since 2015, including regular updates of its drug reimbursement list, price negotiations with drugmakers and national bulk-buy programs. The reforms have greatly reduced the financial burden on patients while improving access to innovative medications. Companies can also refer to this practice when establishing the list of specialty drugs for Huimin Insurance. Extending to the whole PHI market, the government should facilitate market order and effective management, and strengthen the supervision of PHI (62). Companies should improve the reimbursement coverage to build trust with potential customers.

Filing a gap of the literature, this is the first study of preferences for PHI using DCE in China. Modeled on Huimin Insurance, we conducted our PHI study in a city where Huimin Insurance type schemes had not yet been implemented, which avoids preconceptions about the DCE attributes. In addition, attributes and levels, such as premiums and deductibles, were designed using real data based on issued Huimin Insurance policies in other similar cities.

Nevertheless, some limitations remain in our study. First, some attributes were simplified like pre-existing conditions and specialty drugs to ease comprehension by participants. Second, the rural population in the sample was higher than that in Shiyan's official statistics (63). Third, we examined the sociodemographic factors of heterogeneity, but found no correlates with 100% compensation rates. This implies that there may exist additional influences, such as risk attitudes (14), which need further exploration. Fourth, our investigation was specific to the preferences of Shiyan residents. While the results may be illustrative to other parts of China, further studies in other cities are required. Finally, opt-out was excluded to the choice set in order to reduce the complexity of DCE. However, it may less closely resemble an actual situation that participants would confront for PHI (39). Future research should be undertaken in cities that have already implemented Huimin Insurance, adding opt-out and refining the relevant attributes in the experiment design. Similar PHI studies should be undertaken in other areas in China to further explore the applicability of our results.

## Conclusions

This first study to explore preferences of PHI in China fills an important gap in the PHI literature. As a supplement to SHI, private insurers should develop Huimin Insurance with a high reimbursement ratio, expansion of government-involvement, a benefit package with comprehensive healthcare services, compensation for pre-existing conditions, and low premiums and deductibles. The results are considered to contribute to the original design and future improvements of the product from the demand-side in the context of Shiyan. Our findings also provide evidence to guide Huimin Insurance in other regions of China to promote sustainable and healthy development of the PHI market.

## Data availability statement

The raw data supporting the conclusions of this article will be made available by the authors, without undue reservation.

## Ethics statement

Written informed consent was obtained from the individual(s) for the publication of any potentially identifiable images or data included in this article.

## Author contributions

NC and JB: data analysis, interpretation, and drafting the manuscript. SN, EM, and JW: revising the manuscript. JW and JT: study design and supervision. All authors contributed to the article and approved the submitted version.

## Funding

This research was supported by a grant from Taikang Yicai Public Health and Epidemic Control Fund.

## Conflict of interest

The authors declare that the research was conducted in the absence of any commercial or financial relationships that could be construed as a potential conflict of interest.

## Publisher's note

All claims expressed in this article are solely those of the authors and do not necessarily represent those of their affiliated organizations, or those of the publisher, the editors and the reviewers. Any product that may be evaluated in this article, or claim that may be made by its manufacturer, is not guaranteed or endorsed by the publisher.
