# Supplementary material for: Preferences for private health insurance in China: A discrete choice experiment
Source: Front Public Health. 2022 Sep 6;10:985582. doi: 10.3389/fpubh.2022.985582 (PMC9486459; doi:10.3389/fpubh.2022.985582)
Supplement: Supplementary file 1 [file Table_1.docx]

Supplementary Material 1

# The discrete choice experiment questionnaire

Scenario 1

| **Attributes** | **A** | **B** |
| --- | --- | --- |
| Government involvement | Yes | No |
| Premium (CNY per year) | 79 | 159 |
| Benefit package | Out-of-pocket expenses for hospitalization services within social health insurance catalogue + specialty drugs costs outside social health insurance catalogue | Out-of-pocket expenses for hospitalization services within social health insurance catalogue + specialty drugs costs outside social health insurance catalogue + out-of-pocket expenses for hospitalization services not covered by social health insurance |
| Deductible (CNY) | 15,000 | 20,000 |
| Reimbursement ratio | 60% | 80% |
| Compensation for pre-existing conditions | No | Yes |
| **Which one do you prefer?** | **🌕** | **🌕** |

Scenario 2

| **Attributes** | **A** | **B** |
| --- | --- | --- |
| Government involvement | Yes | No |
| Premium (CNY per year) | 199 | 119 |
| Benefit package | Out-of-pocket expenses for hospitalization services within social health insurance catalogue + specialty drugs costs outside social health insurance catalogue + out-of-pocket expenses for hospitalization services not covered by social health insurance | Out-of-pocket expenses for hospitalization services within social health insurance catalogue |
| Deductible (CNY) | 15,000 | 18,000 |
| Reimbursement ratio | 100% | 80% |
| Compensation for pre-existing conditions | Yes | No |
| **Which one do you prefer?** | **🌕** | **🌕** |

Scenario 3

| **Attributes** | **A** | **B** |
| --- | --- | --- |
| Government involvement | No | Yes |
| Premium (CNY per year) | 159 | 119 |
| Benefit package | Out-of-pocket expenses for hospitalization services within social health insurance catalogue | Out-of-pocket expenses for hospitalization services within social health insurance catalogue + specialty drugs costs outside social health insurance catalogue |
| Deductible (CNY) | 18,000 | 20,000 |
| Reimbursement ratio | 60% | 100% |
| Compensation for pre-existing conditions | No | Yes |
| **Which one do you prefer?** | **🌕** | **🌕** |

Scenario 4

| **Attributes** | **A** | **B** |
| --- | --- | --- |
| Government involvement | Yes | No |
| Premium (CNY per year) | 159 | 39 |
| Benefit package | Out-of-pocket expenses for hospitalization services within social health insurance catalogue + specialty drugs costs outside social health insurance catalogue | Out-of-pocket expenses for hospitalization services within social health insurance catalogue |
| Deductible (CNY) | 18,000 | 15,000 |
| Reimbursement ratio | 60% | 100% |
| Compensation for pre-existing conditions | Yes | No |
| **Which one do you prefer?** | **🌕** | **🌕** |

Scenario 5

| **Attributes** | **A** | **B** |
| --- | --- | --- |
| Government involvement | Yes | No |
| Premium (CNY per year) | 119 | 79 |
| Benefit package | Out-of-pocket expenses for hospitalization services within social health insurance catalogue + specialty drugs costs outside social health insurance catalogue + out-of-pocket expenses for hospitalization services not covered by social health insurance | Out-of-pocket expenses for hospitalization services within social health insurance catalogue |
| Deductible (CNY) | 18,000 | 20,000 |
| Reimbursement ratio | 80% | 60% |
| Compensation for pre-existing conditions | No | Yes |
| **Which one do you prefer?** | **🌕** | **🌕** |

Scenario 6

| **Attributes** | **A** | **B** |
| --- | --- | --- |
| Government involvement | Yes | No |
| Premium (CNY per year) | 159 | 119 |
| Benefit package | Out-of-pocket expenses for hospitalization services within social health insurance catalogue + specialty drugs costs outside social health insurance catalogue | Out-of-pocket expenses for hospitalization services within social health insurance catalogue + specialty drugs costs outside social health insurance catalogue + out-of-pocket expenses for hospitalization services not covered by social health insurance |
| Deductible (CNY) | 20,000 | 18,000 |
| Reimbursement ratio | 80% | 60% |
| Compensation for pre-existing conditions | No | Yes |
| **Which one do you prefer?** | **🌕** | **🌕** |

Scenario 7 (Consistency test)

| **Attributes** | **A** | **B** |
| --- | --- | --- |
| Government involvement | No | No |
| Premium (CNY per year) | 119 | 79 |
| Benefit package | Out-of-pocket expenses for hospitalization services within social health insurance catalogue + specialty drugs costs outside social health insurance catalogue | Out-of-pocket expenses for hospitalization services within social health insurance catalogue + specialty drugs costs outside social health insurance catalogue + out-of-pocket expenses for hospitalization services not covered by social health insurance |
| Deductible (CNY) | 18,000 | 15,000 |
| Reimbursement ratio | 80% | 80% |
| Compensation for pre-existing conditions | No | Yes |
| **Which one do you prefer?** | **🌕** | **🌕** |

Scenario 8

| **Attributes** | **A** | **B** |
| --- | --- | --- |
| Government involvement | No | Yes |
| Premium (CNY per year) | 79 | 199 |
| Benefit package | Out-of-pocket expenses for hospitalization services within social health insurance catalogue + specialty drugs costs outside social health insurance catalogue + out-of-pocket expenses for hospitalization services not covered by social health insurance | Out-of-pocket expenses for hospitalization services within social health insurance catalogue |
| Deductible (CNY) | 18,000 | 15,000 |
| Reimbursement ratio | 100% | 80% |
| Compensation for pre-existing conditions | No | Yes |
| **Which one do you prefer?** | **🌕** | **🌕** |

Scenario 9

| **Attributes** | **A** | **B** |
| --- | --- | --- |
| Government involvement | Yes | No |
| Premium (CNY per year) | 119 | 39 |
| Benefit package | Out-of-pocket expenses for hospitalization services within social health insurance catalogue | Out-of-pocket expenses for hospitalization services within social health insurance catalogue + specialty drugs costs outside social health insurance catalogue |
| Deductible (CNY) | 20,000 | 15,000 |
| Reimbursement ratio | 100% | 80% |
| Compensation for pre-existing conditions | No | Yes |
| **Which one do you prefer?** | **🌕** | **🌕** |

Scenario 10

| **Attributes** | **A** | **B** |
| --- | --- | --- |
| Government involvement | Yes | No |
| Premium (CNY per year) | 39 | 119 |
| Benefit package | Out-of-pocket expenses for hospitalization services within social health insurance catalogue + specialty drugs costs outside social health insurance catalogue + out-of-pocket expenses for hospitalization services not covered by social health insurance | Out-of-pocket expenses for hospitalization services within social health insurance catalogue + specialty drugs costs outside social health insurance catalogue |
| Deductible (CNY) | 20,000 | 15,000 |
| Reimbursement ratio | 60% | 100% |
| Compensation for pre-existing conditions | No | Yes |
| **Which one do you prefer?** | **🌕** | **🌕** |

Scenario 11

| **Attributes** | **A** | **B** |
| --- | --- | --- |
| Government involvement | No | Yes |
| Premium (CNY per year) | 199 | 159 |
| Benefit package | Out-of-pocket expenses for hospitalization services within social health insurance catalogue + specialty drugs costs outside social health insurance catalogue | Out-of-pocket expenses for hospitalization services within social health insurance catalogue |
| Deductible (CNY) | 20,000 | 15,000 |
| Reimbursement ratio | 60% | 100% |
| Compensation for pre-existing conditions | No | Yes |
| **Which one do you prefer?** | **🌕** | **🌕** |

Scenario 12

| **Attributes** | **A** | **B** |
| --- | --- | --- |
| Government involvement | Yes | No |
| Premium (CNY per year) | 79 | 159 |
| Benefit package | Out-of-pocket expenses for hospitalization services within social health insurance catalogue | Out-of-pocket expenses for hospitalization services within social health insurance catalogue + specialty drugs costs outside social health insurance catalogue |
| Deductible (CNY) | 18,000 | 15,000 |
| Reimbursement ratio | 80% | 60% |
| Compensation for pre-existing conditions | Yes | No |
| **Which one do you prefer?** | **🌕** | **🌕** |

Scenario 13

| **Attributes** | **A** | **B** |
| --- | --- | --- |
| Government involvement | Yes | No |
| Premium (CNY per year) | 119 | 199 |
| Benefit package | Out-of-pocket expenses for hospitalization services within social health insurance catalogue + specialty drugs costs outside social health insurance catalogue + out-of-pocket expenses for hospitalization services not covered by social health insurance | Out-of-pocket expenses for hospitalization services within social health insurance catalogue |
| Deductible (CNY) | 20,000 | 18,000 |
| Reimbursement ratio | 60% | 100% |
| Compensation for pre-existing conditions | Yes | No |
| **Which one do you prefer?** | **🌕** | **🌕** |

Scenario 14

| **Attributes** | **A** | **B** |
| --- | --- | --- |
| Government involvement | No | Yes |
| Premium (CNY per year) | 79 | 39 |
| Benefit package | Out-of-pocket expenses for hospitalization services within social health insurance catalogue + specialty drugs costs outside social health insurance catalogue + out-of-pocket expenses for hospitalization services not covered by social health insurance | Out-of-pocket expenses for hospitalization services within social health insurance catalogue + specialty drugs costs outside social health insurance catalogue |
| Deductible (CNY) | 15,000 | 18,000 |
| Reimbursement ratio | 80% | 100% |
| Compensation for pre-existing conditions | No | Yes |
| **Which one do you prefer?** | **🌕** | **🌕** |
