# Supplementary material for: Preferences for private health insurance in China: A discrete choice experiment
Source: Front Public Health. 2022 Sep 6;10:985582. doi: 10.3389/fpubh.2022.985582 (PMC9486459; doi:10.3389/fpubh.2022.985582)
Supplement: Supplementary file 2 [file Table_2.docx]

Supplementary Material 2

# Results of subgroup analysis and interaction effects

Table S1 Results of subgroup analysis and interaction effects stratified by sex

| **Attributes** | **Subgroup analysis** | | | | **Interaction effects** |
| --- | --- | --- | --- | --- | --- |
|  | **Male (n=369)** | | **Female (n=578)** | |  |
|  | **Mean (SE)** | **SD (SE)** | **Mean (SE)** | **SD (SE)** | **Mean (SE)** |
| Government involvement |  |  |  |  |  |
| No | Reference | - | Reference | - |  |
| Yes | 0.759*** (0.079) | 1.039*** (0.074) | 0.692*** (0.062) | 0.989*** (0.059) | -0.037 (0.086) |
| Premium, CNY/year | -0.003*** (0.001) | - | -0.004*** (0) | - |  |
| Benefit package |  |  |  |  |  |
| Basic | Reference | - | Reference | - |  |
| Expanded 1 | 0.616*** (0.06) | 0.017 (0.112) | 0.702*** (0.049) | -0.087 (0.165) |  |
| Expanded 2 | 0.938*** (0.074) | 0.424*** (0.095) | 1.097*** (0.065) | 0.635*** (0.068) | 0.142 (0.076) |
| Deductible, CNY |  |  |  |  |  |
| 15,000 | Reference | - | Reference | - |  |
| 18,000 | -0.076 (0.055) | -0.016 (0.111) | 0.043 (0.044) | -0.015 (0.128) |  |
| 20,000 | -0.111 (0.059) | 0.044 (0.28) | -0.086 (0.049) | 0.277* (0.109) |  |
| Reimbursement ratio, % |  |  |  |  |  |
| 60 | Reference | - | Reference | - |  |
| 80 | 0.693*** (0.058) | -0.032 (0.24) | 0.657*** (0.047) | 0.008 (0.078) |  |
| 100 | 1.295*** (0.081) | 0.915*** (0.081) | 1.241*** (0.066) | 0.917*** (0.066) | -0.063 (0.091) |
| Compensation for pre-existing conditions |  |  |  |  |  |
| No | Reference | - | Reference | - |  |
| Yes | 0.425*** (0.061) | 0.644*** (0.063) | 0.469*** (0.049) | 0.628*** (0.052) | 0.052 (0.068) |

SE, standard error; SD, standard deviation; CNY, Chinese Yuan.

***p < 0.001, **p < 0.01, *p < 0.05.

Table S2 Results of subgroup analysis and interaction effects stratified by age

|  | **Subgroup analysis** | | | | **Interaction effects** |
| --- | --- | --- | --- | --- | --- |
| **Attributes** | **Age 18-59 (n=883)** | | **Age 60-75 (n=64)** | |  |
|  | **Mean (SE)** | **SD (SE)** | **Mean (SE)** | **SD (SE)** | **Mean (SE)** |
| Government involvement |  |  |  |  |  |
| No | Reference | - | Reference | - |  |
| Yes | 0.701*** (0.050) | 0.983*** (0.047) | 1.031*** (0.243) | 1.331*** (0.214) | 0.082 (0.168) |
| Premium, CNY/year | -0.004*** (0.000) | - | -0.002 (0.001) | - |  |
| Benefit package |  |  |  |  |  |
| Basic | Reference | - | Reference | - |  |
| Expanded 1 | 0.676*** (0.039) | -0.044 (0.111) | 0.524** (0.152) | -0.013 (0.278) |  |
| Expanded 2 | 1.023*** (0.051) | 0.569*** (0.056) | 1.149*** (0.199) | 0.492* (0.230) | -0.009 (0.149) |
| Deductible, CNY |  |  |  |  |  |
| 15,000 | Reference | - | Reference | - |  |
| 18,000 | -0.009 (0.036) | -0.031 (0.079) | 0.091 (0.148) | 0.299 (0.263) |  |
| 20,000 | -0.104** (0.039) | 0.162 (0.126) | 0.053 (0.168) | -0.533* (0.249) |  |
| Reimbursement ratio, % |  |  |  |  |  |
| 60 | Reference | - | Reference | - |  |
| 80 | 0.673*** (0.038) | 0.001 (0.073) | 0.621*** (0.149) | -0.087 (0.386) |  |
| 100 | 1.256*** (0.052) | 0.875*** (0.052) | 1.317*** (0.249) | 1.425*** (0.251) | -0.045 (0.174) |
| Compensation for pre-existing conditions |  |  |  |  |  |
| No | Reference | - | Reference | - |  |
| Yes | 0.460*** (0.039) | 0.639*** (0.042) | 0.337* (0.144) | 0.533** (0.170) | -0.089 (0.132) |

SE, standard error; SD, standard deviation; CNY, Chinese Yuan.

***p < 0.001, **p < 0.01, *p < 0.05.

Table S3 Results of subgroup analysis and interaction effects stratified by residence type

| **Attributes** | **Subgroup analysis** | | | | **Interaction effects** |
| --- | --- | --- | --- | --- | --- |
|  | **Rural (n=163)** | | **Urban (n=784)** | |  |
|  | **Mean (SE)** | **SD (SE)** | **Mean (SE)** | **SD (SE)** | **Mean (SE)** |
| Government involvement |  |  |  |  |  |
| No | Reference | - | Reference | - |  |
| Yes | 0.665*** (0.102) | 0.816*** (0.096) | 0.735*** (0.056) | 1.057*** (0.053) | 0.085 (0.111) |
| Premium, CNY/year | -0.007*** (0.001) | - | -0.003*** (0) | - |  |
| Benefit package |  |  |  |  |  |
| Basic | Reference | - | Reference | - |  |
| Expanded 1 | 0.299*** (0.083) | 0.025 (0.114) | 0.766*** (0.043) | -0.064 (0.194) |  |
| Expanded 2 | 0.672*** (0.105) | 0.542*** (0.12) | 1.136*** (0.056) | 0.578*** (0.061) | 0.142 (0.097) |
| Deductible, CNY |  |  |  |  |  |
| 15,000 | Reference | - | Reference | - |  |
| 18,000 | -0.043 (0.076) | 0.001 (0.13) | -0.003 (0.039) | 0.002 (0.119) |  |
| 20,000 | -0.094 (0.089) | 0.425** (0.143) | -0.106* (0.042) | -0.111 (0.189) |  |
| Reimbursement ratio, % |  |  |  |  |  |
| 60 | Reference | - | Reference | - |  |
| 80 | 0.457*** (0.08) | -0.004 (0.134) | 0.728*** (0.042) | -0.015 (0.088) |  |
| 100 | 0.874*** (0.102) | 0.68*** (0.114) | 1.368*** (0.06) | 0.98*** (0.058) | 0.158 (0.115) |
| Compensation for pre-existing conditions |  |  |  |  |  |
| No | Reference | - | Reference | - |  |
| Yes | 0.25** (0.075) | 0.366*** (0.097) | 0.505*** (0.044) | 0.702*** (0.047) | 0.197*(0.087) |

SE, standard error; SD, standard deviation; CNY, Chinese Yuan.

***p < 0.001, **p < 0.01, *p < 0.05.

Table S4 Results of subgroup analysis and interaction effects stratified by marital status

| **Attributes** | **Subgroup analysis** | | | | **Interaction effects** |
| --- | --- | --- | --- | --- | --- |
|  | **Widowed, unmarried, or divorced (n=162)** | | **Married (n=785)** | |  |
|  | **Mean (SE)** | **SD (SE)** | **Mean (SE)** | **SD (SE)** | **Mean (SE)** |
| Government involvement |  |  |  |  |  |
| No | Reference | - | Reference | - |  |
| Yes | 0.744*** (0.12) | 1.017*** (0.116) | 0.711*** (0.053) | 0.998*** (0.05) | -0.008 (0.111) |
| Premium, CNY/year | -0.004*** (0.001) | - | -0.003*** (0) | - |  |
| Benefit package |  |  |  |  |  |
| Basic | Reference | - | Reference | - |  |
| Expanded 1 | 0.623*** (0.093) | 0.117 (0.213) | 0.673*** (0.041) | -0.034 (0.115) |  |
| Expanded 2 | 1.025*** (0.123) | 0.633*** (0.135) | 1.03*** (0.053) | 0.54*** (0.06) | -0.01 (0.099) |
| Deductible, CNY |  |  |  |  |  |
| 15,000 | Reference | - | Reference | - |  |
| 18,000 | -0.09 (0.084) | -0.014 (0.202) | 0.014 (0.038) | -0.017 (0.089) |  |
| 20,000 | -0.107 (0.096) | -0.392* (0.162) | -0.094* (0.041) | 0.147 (0.147) |  |
| Reimbursement ratio, % |  |  |  |  |  |
| 60 | Reference | - | Reference | - |  |
| 80 | 0.713*** (0.091) | 0.045 (0.253) | 0.66*** (0.04) | -0.011 (0.072) |  |
| 100 | 1.18*** (0.13) | 1*** (0.125) | 1.272*** (0.055) | 0.884*** (0.055) | 0.205 (0.117) |
| Compensation for pre-existing conditions |  |  |  |  |  |
| No | Reference | - | Reference | - |  |
| Yes | 0.493*** (0.093) | 0.638*** (0.101) | 0.442*** (0.041) | 0.632*** (0.045) | -0.024 (0.088) |

SE, standard error; SD, standard deviation; CNY, Chinese Yuan.

***p < 0.001, **p < 0.01, *p < 0.05.

Table S5 Results of subgroup analysis and interaction effects stratified by education level

| **Attributes** | **Subgroup analysis** | | | | **Interaction effects** |
| --- | --- | --- | --- | --- | --- |
|  | **Uneducated (<6 y) (n=121)** | | **Educated (≥6 y) (n=826)** | |  |
|  | **Mean (SE)** | **SD (SE)** | **Mean (SE)** | **SD (SE)** | **Mean (SE)** |
| Government involvement |  |  |  |  |  |
| No | Reference | - | Reference | - |  |
| Yes | 0.523*** (0.101) | 0.566*** (0.101) | 0.764*** (0.056) | 1.1*** (0.054) | 0.317* (0.124) |
| Premium, CNY/year | -0.007*** (0.001) | - | -0.003*** (0) | - |  |
| Benefit package |  |  |  |  |  |
| Basic | Reference | - | Reference | - |  |
| Expanded 1 | 0.294** (0.092) | 0.018 (0.131) | 0.751*** (0.043) | 0.128 (0.12) |  |
| Expanded 2 | 0.631*** (0.114) | 0.46** (0.138) | 1.134*** (0.056) | 0.605*** (0.06) | 0.183 (0.11) |
| Deductible, CNY |  |  |  |  |  |
| 15,000 | Reference | - | Reference | - |  |
| 18,000 | 0.097 (0.087) | -0.01 (0.141) | -0.032 (0.038) | -0.039 (0.095) |  |
| 20,000 | -0.079 (0.092) | -0.001 (0.185) | -0.107* (0.043) | -0.315*** (0.086) |  |
| Reimbursement ratio, % |  |  |  |  |  |
| 60 | Reference | - | Reference | - |  |
| 80 | 0.315*** (0.089) | 0.019 (0.163) | 0.749*** (0.041) | -0.007 (0.081) |  |
| 100 | 0.777*** (0.122) | 0.812*** (0.132) | 1.378*** (0.058) | 0.945*** (0.056) | 0.179 (0.13) |
| Compensation for pre-existing conditions |  |  |  |  |  |
| No | Reference | - | Reference | - |  |
| Yes | 0.208* (0.087) | 0.453*** (0.104) | 0.497*** (0.043) | 0.681*** (0.045) | 0.187 (0.097) |

SE, standard error; SD, standard deviation; CNY, Chinese Yuan.

***p < 0.001, **p < 0.01, *p < 0.05.

Table S6 Results of subgroup analysis and interaction effects stratified by chronic conditions

| **Attributes** | **Subgroup analysis** | | | | **Interaction effects** |
| --- | --- | --- | --- | --- | --- |
|  | **None (n=717)** | | **One of more (n=196)** | |  |
|  | **Mean (SE)** | **SD (SE)** | **Mean (SE)** | **SD (SE)** | **Mean (SE)** |
| Government involvement |  |  |  |  |  |
| No | Reference | - | Reference | - |  |
| Yes | 0.698*** (0.057) | 1.031*** (0.054) | 0.749*** (0.104) | 0.915*** (0.097) | -0.112 (0.102) |
| Premium, CNY/year | -0.003*** (0) | - | -0.004*** (0.001) | - |  |
| Benefit package |  |  |  |  |  |
| Basic | Reference | - | Reference | - |  |
| Expanded 1 | 0.714*** (0.044) | 0.045 (0.137) | 0.578*** (0.083) | -0.044 (0.252) |  |
| Expanded 2 | 1.058*** (0.058) | 0.613*** (0.06) | 1.028*** (0.106) | -0.531*** (0.118) | -0.076 (0.091) |
| Deductible, CNY |  |  |  |  |  |
| 15,000 | Reference | - | Reference | - |  |
| 18,000 | 0.019 (0.04) | 0.011 (0.104) | -0.096 (0.077) | 0.036 (0.146) |  |
| 20,000 | -0.067 (0.044) | -0.235* (0.113) | -0.19* (0.084) | -0.206 (0.234) |  |
| Reimbursement ratio, % |  |  |  |  |  |
| 60 | Reference | - | Reference | - |  |
| 80 | 0.717*** (0.043) | -0.022 (0.083) | 0.576*** (0.079) | -0.064 (0.163) |  |
| 100 | 1.312*** (0.058) | 0.872*** (0.058) | 1.23*** (0.124) | 1.104*** (0.122) | -0.009 (0.107) |
| Compensation for pre-existing conditions |  |  |  |  |  |
| No | Reference | - | Reference | - |  |
| Yes | 0.448*** (0.043) | 0.602*** (0.047) | 0.474*** (0.089) | 0.783*** (0.094) | 0.088 (0.081) |

SE, standard error; SD, standard deviation; CNY, Chinese Yuan.

***p < 0.001, **p < 0.01, *p < 0.05.

Table S7 Results of subgroup analysis and interaction effects stratified by yearly income

| **Attributes** | **Subgroup analysis** | | | | **Interaction effects** |
| --- | --- | --- | --- | --- | --- |
|  | **Lower than Shiyan average (n=391)** | | **Higher/equal Shiyan average (556)** | |  |
|  | **Mean (SE)** | **SD (SE)** | **Mean (SE)** | **SD (SE)** | **Mean (SE)** |
| Government involvement |  |  |  |  |  |
| No | Reference | - | Reference | - |  |
| Yes | 0.683*** (0.072) | 0.953*** (0.069) | 0.749*** (0.066) | 1.056*** (0.063) | 0.067 (0.085) |
| Premium, CNY/year | -0.005*** (0) | - | -0.003*** (0) | - |  |
| Benefit package |  |  |  |  |  |
| Basic | Reference | - | Reference | - |  |
| Expanded 1 | 0.551*** (0.056) | -0.061 (0.186) | 0.761*** (0.052) | 0.003 (0.126) |  |
| Expanded 2 | 0.829*** (0.072) | 0.561*** (0.08) | 1.195*** (0.068) | 0.555*** (0.072) | 0.202** (0.075) |
| Deductible, CNY |  |  |  |  |  |
| 15,000 | Reference | - | Reference | - |  |
| 18,000 | 0.051 (0.052) | 0.012 (0.113) | -0.048 (0.046) | -0.028 (0.119) |  |
| 20,000 | -0.157** (0.056) | -0.104 (0.251) | -0.051 (0.051) | -0.215 (0.149) |  |
| Reimbursement ratio, % |  |  |  |  |  |
| 60 | Reference | - | Reference | - |  |
| 80 | 0.494*** (0.054) | 0.014 (0.103) | 0.803*** (0.05) | 0.009 (0.099) |  |
| 100 | 1.112*** (0.074) | 0.818*** (0.077) | 1.376*** (0.071) | 0.981*** (0.068) | 0.012 (0.09) |
| Compensation for pre-existing conditions |  |  |  |  |  |
| No | Reference | - | Reference | - |  |
| Yes | 0.373*** (0.057) | 0.619*** (0.06) | 0.506*** (0.051) | 0.64*** (0.055) | 0.082 (0.067) |

SE, standard error; SD, standard deviation; CNY, Chinese Yuan.

***p < 0.001, **p < 0.01, *p < 0.05.

Table S8 Regression results of subgroup analysis and interaction effects stratified by social health insurance

| **Attributes** | **Subgroup analysis** | | | | **Interaction effects** |
| --- | --- | --- | --- | --- | --- |
|  | **URRBMI (n=366)** | | **UEBMI (n=571)** | |  |
|  | **Mean (SE)** | **SD (SE)** | **Mean (SE)** | **SD (SE)** | **Mean (SE)** |
| Government involvement |  |  |  |  |  |
| No | Reference | - | Reference | - |  |
| Yes | 0.663*** (0.07) | 0.828*** (0.066) | 0.772*** (0.069) | 1.149*** (0.065) | 0.176* (0.085) |
| Premium, CNY/year | -0.005*** (0) | - | -0.002*** (0) |  |  |
| Benefit package |  |  | - |  |  |
| Basic | Reference | - | Reference | - |  |
| Expanded 1 | 0.464*** (0.057) | 0.013 (0.11) | 0.836*** (0.052) | -0.024 (0.23) |  |
| Expanded 2 | 0.836*** (0.072) | 0.502*** (0.083) | 1.225*** (0.069) | 0.62*** (0.073) | 0.171* (0.076) |
| Deductible, CNY |  |  |  |  |  |
| 15,000 | Reference | - | Reference | - |  |
| 18,000 | 0.012 (0.052) | 0.001 (0.131) | -0.03 (0.047) | 0.021 (0.109) |  |
| 20,000 | -0.117* (0.058) | -0.212 (0.147) | -0.106* (0.051) | -0.236 (0.126) |  |
| Reimbursement ratio, % |  |  |  |  |  |
| 60 | Reference | - | Reference | - |  |
| 80 | 0.51*** (0.055) | -0.017 (0.105) | 0.811*** (0.051) | 0.005 (0.103) |  |
| 100 | 1.035*** (0.074) | 0.804*** (0.078) | 1.448*** (0.073) | 1.003*** (0.069) | 0.037 (0.09) |
| Compensation for pre-existing conditions |  |  |  |  |  |
| No | Reference | - | Reference | - |  |
| Yes | 0.337*** (0.057) | 0.607*** (0.062) | 0.54*** (0.053) | 0.694*** (0.055) | 0.05 (0.068) |

URRBMI, Urban-Rural Residents Basic Medical Insurance; UEBMI, Urban Employee Basic Medical Insurance; SE, standard error; SD, standard deviation; CNY Chinese Yuan.

***p < 0.001, **p < 0.01, *p < 0.05.

Table S9 Results of subgroup analysis stratified and interaction effects by other private health insurance

| **Attributes** | **Subgroup analysis** | | | | **Interaction effects** |
| --- | --- | --- | --- | --- | --- |
|  | **No (n=654)** | | **Yes (n=293)** | |  |
|  | **Mean (SE)** | **SD (SE)** | **Mean (SE)** | **SD (SE)** | **Mean (SE)** |
| Government involvement |  |  |  |  |  |
| No | Reference | - | Reference | - |  |
| Yes | 0.71*** (0.056) | 0.958*** (0.053) | 0.786*** (0.104) | 1.204*** (0.103) | 0.02 (0.091) |
| Premium, CNY/year | -0.004*** (0) | - | -0.003*** (0.001) | - |  |
| Benefit package |  |  |  |  |  |
| Basic | Reference | - | Reference | - |  |
| Expanded 1 | 0.547*** (0.043) | -0.011 (0.093) | 1.042*** (0.089) | 0.358** (0.138) |  |
| Expanded 2 | 0.901*** (0.055) | 0.477*** (0.065) | 1.451*** (0.117) | 0.869*** (0.117) | 0.117 (0.081) |
| Deductible, CNY |  |  |  |  |  |
| 15,000 | Reference | - | Reference | - |  |
| 18,000 | 0.007 (0.04) | 0.003 (0.084) | -0.047 (0.071) | 0.161 (0.233) |  |
| 20,000 | -0.093* (0.043) | -0.083 (0.244) | -0.113 (0.081) | 0.482*** (0.13) |  |
| Reimbursement ratio, % |  |  |  |  |  |
| 60 | Reference | - | Reference | - |  |
| 80 | 0.604*** (0.042) | -0.002 (0.081) | 0.899*** (0.081) | 0.114 (0.19) |  |
| 100 | 1.117*** (0.058) | 0.873*** (0.059) | 1.728*** (0.119) | 1.068*** (0.112) | 0.182 (0.096) |
| Compensation for pre-existing conditions |  |  |  |  |  |
| No | Reference | - | Reference | - |  |
| Yes | 0.385*** (0.043) | 0.587*** (0.047) | 0.631*** (0.081) | 0.794*** (0.088) | 0.182* (0.072) |

SE, standard error; SD, standard deviation; CNY, Chinese Yuan.

***p < 0.001, **p < 0.01, *p < 0.05.
